# Causal effect of chemotherapy received dose intensity on survival outcome: a retrospective study in osteosarcoma

**DOI:** 10.1186/s12874-024-02416-x

**Published:** 2024-12-03

**Authors:** Marta Spreafico, Francesca Ieva, Marta Fiocco

**Affiliations:** 1 Mathematical Institute, Leiden University, Leiden, 2333 CC The Netherlands; 2 Department of Biomedical Data Sciences, Leiden University Medical Center, Leiden, 2333 ZA The Netherlands; 3 MOX – Department of Mathematics, Politecnico di Milano, Milan, 20133 Italy; 4 Health Data Science Center, Human Technopole, Milan, 20157 Italy; 5 Trial and Data Center, Princess Máxima Center for Pediatric Oncology, Utrecht, 3584 CS The Netherlands

**Keywords:** Marginal structural Cox models, Inverse Probability of treatment weighting, Effect modification, Target trial emulation, Received dose intensity, Chemotherapy, Toxicity, Event-free survival

## Abstract

**Background:**

This study aims to analyse the effects of reducing Received Dose Intensity (RDI) in chemotherapy treatment for osteosarcoma patients on their survival by using a novel approach. Previous research has highlighted discrepancies between planned and actual RDI, even among patients randomized to the same treatment regimen. To mitigate toxic side effects, treatment adjustments, such as dose reduction or delayed courses, are necessary. Toxicities are therefore risk factors for mortality and predictors of future exposure levels. Toxicity introduces post-assignment confounding when assessing the causal effect of chemotherapy RDI on survival outcomes, a topic of ongoing debate.

**Methods:**

Chemotherapy administration data from BO03 and BO06 Randomized Clinical Trials (RCTs) in ostosarcoma are employed to emulate a target trial with three RDI-based exposure strategies: 1) *standard*, 2) *reduced*, and 3) *highly-reduced* RDI. Investigations are conducted between subgroups of patients characterised by poor or good Histological Responses (HRe), i.e., the strongest known prognostic factor for survival in osteosarcoma. Inverse Probability of Treatment Weighting (IPTW) is first used to transform the original population into a pseudo-population which mimics the target randomized cohort. Then, a Marginal Structural Cox Model with effect modification is employed. Conditional Average Treatment Effects (CATEs) are ultimately measured as the difference between the Restricted Mean Survival Time of *reduced/highly-reduced* RDI strategy and the *standard* one. Confidence Intervals for CATEs are obtained using a novel IPTW-based bootstrap procedure.

**Results:**

Significant effect modifications based on HRe were found. Increasing RDI-reductions led to contrasting trends for poor and good responders: the higher the reduction, the better (worsen) was the survival in poor (good) reponders. Due to their intrinsic resistance to chemotherapy, poor reponders could benefit from reduced RDI, with an average gain of 10.2 and 15.4 months at 5-year for *reduced* and *highly-reduced* exposures, respectively.

**Conclusions:**

This study introduces a novel approach to (i) comprehensively address the challenges related to the analysis of chemotherapy data, (ii) mitigate the *toxicity-treatment-adjustment* bias, and (iii) repurpose existing RCT data for retrospective analyses extending beyond the original trials’ intended scopes.

**Supplementary Information:**

The online version contains supplementary material available at 10.1186/s12874-024-02416-x.

## Background

Osteosarcoma is a rare malignant bone tumor primarily affecting children, adolescents, and young adults, with an annual incidence of 3–4 patients per million [1]. While multidisciplinary management, including neoadjuvant and adjuvant chemotherapy with aggressive surgical resection [2], has improved clinical outcomes, there has been little progress in survival over the past 40 years [3]. The strongest known prognostic factor for both event-free survival (i.e., time to local recurrence, metastatic disease, second malignancy, or death) and overall survival (i.e., time to death) in osteosarcoma is Histological Response (HRe) [4], i.e., the result of the histopathological examination to assess the improvement in microscopic tissue appearance following pre-operative chemotherapy. However, the impact of interventions in chemotherapy dosage and timing on patient survival remains unclear [5]. In this study the primary research questions are:

*Does reduced chemotherapy dose intensity lead to improved Event-Free Survival (EFS) in patients with osteosarcoma who have completed treatment? Does this effect vary among subjects characterized by different histological responses?*

Addressing these questions is very challenging, even with data from Randomized Clinical Trials (RCTs). A first attempt was made in [6], where the authors investigated an Intention-To-Treat (ITT) landmark Cox model including as covariates the planned regimen, HRe, and their interaction. The ITT principle, widely applied in RCTs, measures the effect of *assigning* patients to different regimens [7, 8], disregarding post-randomization events, such as non-adherence or protocol deviations. However, the intensity of the assigned regimen often differs from the intensity of the received dose and actual duration. Interventions and discontinuation in treatment administration are common in real clinical practice, due to the toxic side effects developed by patients over therapy [9] which affect subsequent exposure by delaying the next cycle or reducing chemotherapy doses [10, 11]. This implies that, even if assigned to the same protocol regimen and subjected to all treatment cycles, patients usually receive different drug doses and experience different treatment durations. Being at the same time risk factors for mortality and predictors of future exposure levels, toxicities are *post-assignment confounders* for the effect of received dose intensity on patient’s survival.

To measure the discrepancies between *assigned* (or planned) and *received* (or actual) treatments in terms of both dose reduction and delays, the so-called Received Dose Intensity (RDI) indicator has been introduced [12]. Previous studies showed that there is a mismatch between planned and achieved chemotherapy-RDI in osteosarcoma [5, 11]. Even patients assigned to the same regimen reported substantial variability in RDI at the end of treatment [5, 11, 13]. To evaluate the impact of actually *receiving* a treatment, per-protocol or as-treated analyses can be employed. The first focuses on participants who strictly adhered to the assigned protocol and excludes non-adherent data, while the second considers treatment actually received by patients, regardless of adherence to randomization [8]. Nonetheless, both approaches compromise the balance between patient groups achieved through randomization, potentially introducing selection bias and confounding into the treatment effect estimate. In the presence of confounders, classical survival approaches [14–16] fail to estimate consistent causal effects. An alternative framework that emulates randomization, where confounders (e.g., toxicities) no longer predict treatment, is hence necessary.

In clinical trials, interventions in treatment administration, as well as their underlying reasons, are typically well documented as required by protocols. This existing wealth of information has the potential to be repurposed for additional retrospective analyses beyond the scope of the original RCTs that generated the data, opening up new possibilities for further investigations. More specifically, chemotherapy administration data can be employed to emulate another hypothetical RCT or Target Trial (TT) that explores new research questions on chemotherapy treatment outside the original scope. TT emulation has been introduced in [17] as a method for enabling the application of causal inference methods using observational data. A proper emulation requires a detailed specification of all the necessary protocol components (i.e., eligibility criteria, treatment strategies, treatment assignment, start and end of follow-up, outcomes, causal contrasts or estimands) and a data-analysis plan. This approach is particularly valuable for studying treatments or interventions where randomization is not possible or practical or is no longer present.

### Objectives

In this article, a novel TT emulation based on RCT data of chemotherapy administration with interventions is proposed to estimate the effects of different received exposure strategies on EFS in patients with osteosarcoma aged 40 years or less at baseline. Three exposure strategies are defined and considered: 1) *standard*, 2) *reduced*, and 3) *highly-reduced* RDI. Data from two RCTs in osteosarcoma, namely, the European Osteosarcoma Intergroup (EOI) studies BO03 [18] and BO06 [6] (European Organisation for Research and Treatment of Cancer EORTC 80861 and 80931, respectively) are analysed. By considering patients who successfully completed the six cycles of the same chemotherapy regimen, it is shown how properly documented chemotherapy-administration data can be reused to address novel research questions.

A Marginal Structural Cox Model (Cox MSM) with effect modification estimated by using Inverse Probability of Treatment Weighting (IPTW) [19] is employed to study a model similar to the ITT landmark Cox landmark model in [6] in a causal setting. Specifically, the planned regimen in [6] is replaced with our RDI-exposure strategies and their effect is supposed to vary based on the HRe (i.e., the effect modifier). IPTW is used to mimic randomization in the defined TT, where RDI-exposure is no longer confounded by toxicities or other confounders, so that a crude analysis suffices to estimate the effectiveness of RDI-reduction exposures on EFS in both HRe sub-groups. Conditional Average Treatment Effects (CATEs) are finally measured as the difference between the Restricted Mean Survival Time (RMST) of *reduced/highly-reduced* RDI strategy and the *standard* one. A novel generalized bootstrap procedure [20, 21] utilizing unequal IPTW-based probability sampling [22, 23] and preserving the sizes of the sub-cohorts defined by different combinations of strategies and effect modifier levels is proposed to compute confidence intervals for CATEs.

The overall procedure hence requires (i) a proper definition of the RDI-exposure strategy, (ii) a tailor-made identification of all possible *pre-assignement* and *post-assignement* confounders, and (iii) a proper characterisation of the causal structure of the chemotherapy data through a Direct Acyclic Graph (DAG) [19, 24]. Furthermore, since adjustments in treatment allocation are determined by the overall toxic burden of each patient, the different types and number of side effects must be adequately summarized and quantified. The new longitudinal Multiple Overall Toxicity (MOTox) score introduced in [25] is hence adapted to the data under study. This allows multiple toxicities to be included within the causal inference framework in a novel way.

The ultimate goal is to introduce an innovative and comprehensive RDI-based analysis of chemotherapy administration data with interventions. A tutorial-like explanations of the challenges inherent in this context is provided along with novel problem-solving strategies. To the best of our knowledge, this study is the first to apply IPTW-based techniques to survival RCT data, aiming to mitigate the *toxicity-treatment-adjustment* bias when estimating the effects of RDI reductions on EFS, while considering intrinsic personal responses to chemotherapy. Source code for the current study is available here: https://github.com/mspreafico/TTEcausalRDI.

## Methods

### Data sources description: RCT data with interventions

Data from control arms of European Osteosarcoma Intergroup (EOI) randomised clinical trials (RCT) BO03 and BO06 (EORTC 80861 and 80931, respectively) were analysed. Patients aged 40 years or less with a histologically confirmed diagnosis of high-grade nonmetastatic osteosarcoma of the extremity were included in the trials. In both trials, control arms were characterized by the standard EOI treatment structured in 6 cycles of 3-weekly Cisplatin (CDDP) (100 $mg/m^2$) plus Doxorubicin (DOX) (75 $mg/m^2$), and compared to a different therapy regimen (i.e., variant of Rosen’s T10 regimen in BO03 [26] and a 2-weekly intensified version of CDDP+DOX in BO06 [6]). Results about the primary analyses on BO03 and BO06 data can be found in [6, 18].

**Fig. 1:**
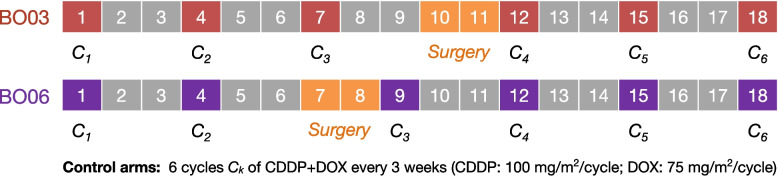
Control arms design for BO03 and B006 randomised clinical trials, characterized by the standard European Osteosarcoma Intergroup treatment structured in 6 cycles of 3-weekly Cisplatin (CDDP) (100 $mg/m^2$) plus Doxorubicin (DOX) (75 $mg/m^2$)

As the control arms design in Fig. 1 shows, in both trials chemotherapy was administered before and after surgical removal of the primary osteosarcoma. At the end of the pre-operative treatment, with a nominal duration of 3 cycles in BO03 and 2 in BO06, the tumour was surgically resected and the level of tumour necrosis – defined as the proportion of the tumor that underwent cell death (necrosis) following pre-operative chemotherapy compared to the entire tumor mass in the resected specimen – was evaluated. Patients were subsequently classified as poor or good responders based on the Histological Response (HRe) assessed in the resected specimen. A good HRe was defined as a percentage of tumor necrosis greater than or equal to 90%; otherwise, HRe was considered poor. Post-operative chemotherapy was intended to resume 2 weeks after surgery.

Along with patients baseline characteristics at randomization (age, gender, allocated chemotherapy regimen, site and location of the tumour), treatment-related variables (administered dose of chemotherapy, cycles timing, haematological parameters, chemotherapy-induced toxicity and histological response to pre-operative chemotherapy) were collected prospectively during therapy. These data provide insights into interventions made during therapy administration (i.e., cycle delays or dose reductions) and the associated toxicity reasons, that led the patient to deviate from the originally planned EOI chemotherapy regimen.

#### Toxicity-driven interventions

As it often occurs in clinical trials, therapy administration was complicated by the need for dynamic adjustments based on the patient’s multi-systemic side effects (e.g., organ toxicity or myelosuppression) developed over time. Toxicities are a threat to patient’s life and must be controlled by either allocating dose reductions/discontinuations or delaying the subsequent course [10].

Toxic side effects were recorded using the Common Terminology Criteria for Adverse Events Version 3 (CTCAE v3.0) [27], with grades ranging from 0 (none) to 4 (life-threatening) (see Supplementary Material A for further details). Toxicities were collected longitudinally in BO06 trial, whereas in BO03 only the highest CTCAE grade (i.e., the most severe) was recorded for each toxicity in both the pre-operative and post-operative periods. According to protocols, the following side effects were linked to specific dose reduction or delay rules: *leucopenia*, *thrombocytopenia*, *oral mucositis*, *ototoxicity*, *cardiotoxicity* and *neurotoxicity*. If different *rule-specific* conditions co-existed and more than one dose reduction (or cumulative delays) applied, the lowest dose (or the highest delays) calculated was employed. According to expert knowledge, although not directly related to a specific adjustment rule, patient’s *generic* conditions of *nausea/vomiting* and *infections* was also taken into account during therapy.

Interventions in treatment administration were hence determined as a combination of overall toxic burden related to both rule-specific and generic conditions. Toxicities impact patient’s survival, leading to a complex post-assignment confounding mechanisms between received chemotherapy dose intensity and the outcome.

#### Assessing interventions through received dose intensity

The so-called Received Dose Intensity (RDI) approach [5, 12, 18] can be adopted to evaluate both dose reductions/discontinuations, time-delays, and their impact in reducing the intensity over the whole therapy. This method summarizes information on treatment interventions by considering both received dose and actual timing. For each patient $i\in \{1,\dots ,N\}$, RDI is defined as the ratio between standardized dose $\Delta _{i}$ and standardized time $\Gamma _{i}$, as follows:

1 $$\begin{aligned} RDI_{i} = \frac{\Delta _i}{\Gamma _i}. \end{aligned}$$

Numerator in (1) represents the *standardized dose*, given by

2 $$\begin{aligned} \Delta _{i} = \frac{1}{2} \left( \Delta _{i}^{CDDP} +\Delta _{i}^{DOX} \right) = \frac{1}{12} \left( \sum _{j=1}^{6} \delta _{ij}^{CDDP} + \sum _{j=1}^{6} \delta _{ij}^{DOX} \right) , \end{aligned}$$

where 6 is the total number of cycles in the EOI regimen, and $\delta _{ij}^{d}$ is the *cycle-standardized received dose* defined as the ratio between the actual dose $[mg/m^2]$ of drug $d \in \{\text {CDDP, DOX}\}$ assumed at cycle *j* and the anticipated dose of drug *d* (CDDP: 100 $mg/m^2$; DOX: 75 $mg/m^2$). Specifically, $\Delta _i<1$ indicates dose-reduced therapies, whereas $\Delta _i>1$ corresponds to dose-augmented therapies.

Denominator in (1) represents the *standardized time* given by

3 $$\begin{aligned} \Gamma _{i} = \frac{\text {actual treatment time}}{\text {anticipated treatment time}}, \end{aligned}$$

where the *actual treatment time* is the difference in days between the starting date of cycle 1 and the 3rd day after the start of cycle 6 (i.e., end of therapy), and the *anticipated treatment time* is $21 \times 5 + 14 + 3= 122$ days (i.e., 5 cycles lasting 21 days each, 14 days of surgery and 3 days after the start of cycle 6). Specifically, $\Gamma _i>1$ indicates delayed therapies, whereas $\Gamma _i<1$ corresponds to compressed treatments.

In general, $\Delta _i \le 1$ and $\Gamma _i \ge 1$ due to dose reductions and delays, respectively; this implies $RDI_i \le 1$. Based on expert knowledge, a RDI (in percentage) of at least 85% is defined as *standard* intensity level, from 85% to 70% is considered *reduced*, whereas below 70% is *highly-reduced*.

### Study design

To address the research questions, a target trial emulation approach is employed [17]. The protocol of the hypothetical TT and its emulation with chemotherapy administration data from BO03/BO06 RCTs are described in Table 1. To be eligible, subjects have to be aged 40 years or less at baseline with a confirmed diagnosis of osteosarcoma. Further inclusion and exclusion criteria are applied to focus on the eligible cohorts of the original BO03/BO06 RCTs. The TT compares three target strategies: 1) *standard*, 2) *reduced*, and 3) *highly-reduced* RDI of the EOI control regimen given by 6 cycles of 3-weekly CDDP+DOX. The final aim is to study the effect, if any, of reductions in RDI (compared to standard) on EFS in subgroups of patients characterized by different HRe. Given that histopathological examination is evaluated after TT randomization, the statistical analysis has to be conducted utilizing a landmark approach [28–30] to appropriately incorporate HRe into the survival model. Specifically, an ITT landmark Cox model, with the landmark point at the time of surgery, is intended to serve as the survival model in the TT to estimate the effects of reduced exposures across HRe levels.

**Table 1:** Outline of the target trial protocol: specification and emulation using RCT data with interventions

Protocol components	Target trial	RCT data with interventions
	Specification	Emulation *[Required data]*
Aim	To estimate the effect, if any, of reductions in received dose intensity on event-free survival across levels of histological response in patients with osteosarcoma aged 40 years or less.	Same as for TT specification.
Eligibility criteria	Patients aged 40 years or less with a histologically confirmed diagnosis of high-grade nonmetastatic osteosarcoma in an extremity long bone.^a^ Patients need to commence chemotherapy within 28 days after biopsy, with normal leukocyte ($\ge 3.5\times 10^9/L$) and platelet ($\ge 100\times 10^9/L$) counts.	Same as for TT specification. *[Age, diagnosis type, start date of therapy, leukocyte count, platelet count]*
Treatment strategies	Chemotherapy dose intensity regimens: *standard*, *reduced*, and *highly-reduced* six-cycle EOI treatment.^b^	Same as for TT specification. *[Received dose, cycle timing]*
Treatment assignments	Eligible persons will be randomly assigned to one strategy and will be aware of which strategy they were assigned to.	Eligible persons assigned to the strategy based on observed dose-intensity behavior at the end of the therapy.
Outcomes	Death, local recurrence, evidence of new or progressive metastatic disease, second malignancy, or a combination of those events.	Same as for TT specification. *[Date of death, local recurrence, or other malignancies]*
Follow-up	Start: treatment assignment. End: death, local recurrence, evidence of new/progressive metastatic disease or second malignancy, or censoring.	Same as for TT specification, except start is the end of therapy. *[Date of loss to follow-up or censoring]*
Causal estimand	Intention-to-treat conditional effects (effect of being assigned to a reduced treatment across subgroups). Per-protocol conditional effect (effect of receiving a reduced treatment as indicated in the protocol across subgroups).	Observational analogue of per-protocol conditional effect across subgroup.
Statistical analysis	Intention-to-treat analysis via landmark Cox model [28–30] to estimate effects of reduced exposures across levels of histological response.^c^	Randomization will be emulated via adjustment for pre-assignment and post-assignment confounders by inverse probability of treatment weighting.
	Conditional average effect measured as contrast of restricted mean survival times over follow-up.	Per-protocol analysis analogous to TT specification. *[Pre-assignement confounders, post-assignment confounders, histological response]*

^a^Ineligible patients: subjects with paraosteal, periosteal, Paget-related, or radiation-induced osteosarcoma; patients with prior malignancy, any chemotherapy before trial entry, reduced glomerular filtration rate ($< 60$mL/min/1.73 m$^2$), cardiac dysfunction, or raised bilirubin

^b^Standard EOI treatment: 6 cycles of 3-weekly CDDP (100$mg/m^2$) plus DOX (75$mg/m^2$)

^c^Samilarly to the ITT landmark Cox model for regimen effect stratified by histological response in [6]

In the cohort selected from the BO03/BO06 data, randomization of target strategies is emulated by adjustment for confounding via IPTW. A pseudo-population is created by weighting each patient based on the inverse probability of observing a specific exposure allocation strategy given the confounders history. The pseudo-population mimics the randomized cohort of the TT and exhibits the following two properties:

i. the pre-assignment and post-assignment history of pseudo-patients no longer predicts exposure to RDI-reductions in the next cycle;
ii. the association between exposure and outcome is the same in both original and pseudo-population.

Therefore, (heterogeneous) causal effects of different exposure strategies (across sub-groups defined by HRe) can be estimated by a crude analysis on the pseudo-population by using a Cox MSM with effect modifications.

### Causal inference framework

To address the research questions at hand, it is imperative to appropriately emulate the target causal inference framework and develop a suitable data analysis plan. This requires both clinical expertise in the treatment of osteosarcoma and statistical knowledge in variable definition and mathematical modeling. Causal analysis involving effect modification focuses on investigating the causal relationship between exposure and outcome across various levels of another factor that impacts this connection, and it requires adjustment for exposure-outcome confounders. The components of our causal framework hence include exposure, outcome, confounders, and effect modifier, as defined in the following sections. The causal structure is finally represented through a Directed Acyclic Graph (DAG) [19, 24]. This process requires special attention to the identifiability assumptions [19, 31] of *consistency*, *no unmeasured confounding*, and *positivity*, discussed in details in Supplementary Material B.

#### Outcome

The endpoint of this study is EFS, defined as time from the end of therapy until the first event (local recurrence, evidence of new or progressive metastatic disease, second malignancy, death, or a combination of those events) or censoring at last contact. Let $T_i$ = $\min (T_i^*,C_i)$ be the observed EFS time, where $T_i^*$ is the true event time, and $C_i$ is the censoring time (i.e., the time from the end of the therapy until the last visit). Let $D_i = I(T_i^* \le C_i)$ be the event indicator (1 when $T_i^* \le C_i$, and 0 otherwise). The EFS outcome for patient $i \in \{1,...,N\}$ is denoted by the pair $(T_i,D_i)$.

#### Exposure

The exposure strategies related to RDI values are now defined based on expert knowledge. A RDI percentage of 85% or more is considered a *standard* intensity level, as reductions up to 15% are classified as negligible. This standard level can be compared to reductions ranging from 15% to 30% (*reduced* intensity) and reductions above 30% (*highly-reduced* intensity). Consequently, covariate $A_i$ for RDI-exposure is defined as a three-level categorical variable, as follows:

4 $$\begin{aligned} A_i = \left\{ \begin{array}{ll} 0 & \text {if}\ RDI_i \ge 0.85\\ 1 & \text {if}\ 0.70 \le RDI_i< 0.85\\ 2 & \text {if} RDI_i < 0.70 \end{array}\right. \end{aligned}$$

that is, $A_i =0$ is equivalent to a *“standard”* RDI, $A_i =1$ to a *“reduced”* RDI, and $A_i =2$ to a *“highly-reduced”* RDI. Accordingly, the three possible treatment/exposure strategies are denoted by $a \in \{0, 1, 2\}$. Based on expert knowledge, these strategies are well-defined to ensure the *consistency* assumption (see Supplementary Material B).

#### Effect modifier

Effect modification focuses on subgroup-specific causal effects of a single type of exposure [19, 32]. In general, a modifying variable *V* should be included into the analysis under two conditions [19]: (i) when the investigators believe that *V* could potentially act as an effect modifier; (ii) when the investigators are more interested in understanding the causal effect of exposure within the groups defined by covariate *V* rather than examining it across the entire population. In the application considered here, variable $V_i$ is the binary covariate representing the HRe of subject *i*, as defined in the original RCTs:

5 $$\begin{aligned} V_i = \left\{ \begin{array}{ll} 0 & \text {if tumour necrosis}_{i} < 90\%\\ 1 & \text {if tumour necrosis}_{i} \ge 90\% \end{array}\right. \end{aligned}$$

that is, $V_i=1$ for patients with a *“good”* HRe, i.e., Good Responders (GRs), while $V_i=0$ denotes patients with a *“poor”* HRe, i.e., Poor Responders (PRs).

#### Confounders

To draw valid conclusions about the causal exposure effect, the set of confounders of the exposure-outcome relationship under study need to be considered in the analysis. According to experts knowledge and protocol guidelines, the following pre-assignment and post-assignment characteristics, denoted by vector $\varvec{L}_i$, satisfy the hypothesis of *no unmeasured confounding* (see Supplementary Material B).

##### Pre-assignment confounders.

Due to their potential influence on drug metabolism and increased toxicity risk, age group, as defined in [33] (*child*: 0–12/0–11 years for males/females; *adolescent*: 13–17/12–16 years for males/females; *adult*: 18/17 or older for males/females), as well as gender (*female*; *male*) serve as pre-assignment confounders. While the trial number (BO03; BO06) does not serve as a significant risk factor for failures (*p*-value of log-rank test for Kaplan-Meier estimators stratified by trial is 0.967 – see Table 2), it can still be considered a pre-assignment confounder, as it reflect the different number of pre-operative cycles (see Fig. 1) and independently predicts dose intensity (*p*-value of chi-squared test for the association between RDI-exposure and trial cohorts is $<0.001$ – see Table 2).

**Table 2:** Patients and trial characteristics

	All	BO03	BO06	
Patients	276	114 (41.3%)	162 (58.7%)	*p*-value^a^
**Age** ^b^				0.259
*Child*	76 (27.5%)	26 (22.8%)	50 (30.9%)	
*Adolescent*	117 (42.4%)	49 (43.0%)	68 (42.0%)	
*Adult*	83 (30.1%)	39 (34.2%)	44 (27.1%)	
**Gender**				0.703
*Female*	109 (39.5%)	43 (37.7%)	66 (40.7%)	
*Male*	167 (60.5%)	71 (62.3%)	96 (59.3%)	
**Rule-specific MOTox** ^c^				
***Pre-operative***				0.009
Median	3.667	3.883	3.500	
IQR	[2.500; 4.500]	[2.667; 4.792]	[2.333; 4.167]	
Min/Max	0/6.167	0/6.167	0/5.833	
***Post-operative***				$<0.001$
Median	4.333	3.833	5.000	
IQR	[3.667; 5.333]	[2.500; 4.958]	[4.000; 5.500]	
Min/Max	0/6.833	0/6.167	0/6.833	
**Generic MOTox** ^d^				
***Pre-operative***				0.017
Median	4.500	4.500	4.000	
IQR	[3.000; 5.500]	[3.500; 5.375]	[3.000; 5.500]	
Min/Max	0/8	0/8	0/7.500	
***Post-operative***				0.021
Median	4.500	4.500	4.000	
IQR	[3.000; 5.500]	[3.500; 5.375]	[3.000; 5.500]	
Min/Max	0/8	0/7.500	0/8	
**Histological Response**				0.732
*Poor*	182 (65.9%)	77 (67.5%)	105 (64.8%)	
*Good*	94 (34.1%)	37 (32.5%)	57 (35.2%)	
**RDI**				$<0.001$
Median	0.759	0.692	0.805	
IQR	[0.649; 0.857]	[0.589; 0.779]	[0.762; 0.899]	
Min/Max	0.376/1.121	0.376/1.028	0.424/1.121	
**RDI exposure**				$<0.001$
*Standard*	75 (27.2%)	10 (8.8%)	65 (40.1%)	
*Reduced*	111 (40.2%)	46 (40.3%)	65 (40.1%)	
*Highly-reduced*	90 (32.6%)	58 (50.9%)	32 (19.8%)	
**EFS status**				0.673
*Censored*	124 (44.9%)	49 (43.0%)	75 (46.3%)	
*With event*	152 (55.1%)	65 (57.0%)	87 (53.7%)	
**EFS time** [months]				0.962
Median^e^ [IQR]	89.59 [50.33; 146.30]			

^a^Categorical variables: *p*-value of chi-squared test for association with the trial. Continuous variables: *p*-value of two-sided Mann-Whitney U test for the variable distribution in BO03 vs BO06 cohort. EFS time: *p*-value of log-rank test for Kaplan-Meier estimators stratified by trial

^b^Age groups were defined according to [33]: *child* (male: 0–12 years; female: 0–11 years), *adolescent* (male: 13–17 years; female: 12–16 years) and *adult* (male: 18 or older; female: age 17 years or older)

^c^Pre-/post-operative MOTox scores computed using Eq. (6) based on *rule-specific* conditions: $\mathcal {M}_{rule} =$ {*leucopenia*, *thrombocytopenia*, *oral mucositis*, *ototoxicity*, *cardiotoxicity*, *neurotoxicity*}

^d^Pre-/post-operative MOTox scores computed using Eq. (6) based on *generic* conditions: $\mathcal {M}_{gen} =$ {*nausea*, *infection*}

^e^Median EFS time was computed using the reverse Kaplan-Meier method [47]

##### Post-assignment confounders.

Conditioning chemotherapy administration over treatment, *rule-specific* and *generic* toxicities are post-assignment confounding factors. To properly address toxicities as confounding covariates, it is essential to accurately quantify and summarize the pre- and post-operative overall toxic burden arising from individual CTCAE side effects. This is achieved by utilizing the new longitudinal Multiple Overall Toxicity (MOTox) score [25]. The MOTox score incorporates three significant components of adverse events: (i) multiple lower-grade chronic toxicities (which may affect the patient’s quality of life); (ii) substantial level in a specific toxicity (potentially causing severe and permanent consequences for the patient); (iii) time dependency.

Since toxicity data over cycles were not recorded for the BO03 trial, MOTox computation is based on pre- and post-operative periods, by considering the highest CTCAE grade recorded for each toxicity during pre/post-operative cycles.

Let $\mathcal {M}_{rule} = \{$*leucopenia*, *thrombocytopenia*, *oral mucositis*, *ototoxicity*, *cardiotoxicity*, *neurotoxicity*$\}$ and $\mathcal {M}_{gen} = \{$*nausea*, *infection*$\}$ be the two disjoint sets of toxicities related to *rule-specific* and *generic* toxicities, respectively. Denote by $k \in \{pre,post\}$ the pre/post-operative time-period. For each patient *i*, let $tox_{ijk}^{m}$ (with value from 0 to 4) be the most severe CTCAE grade of the *m*-th toxicity of type $j \in \{rule,gen\}$ (with $m=1,...,|\mathcal {M}_{j}|$) measured during period *k*. The MOTox score related to set $\mathcal {M}_{j}$ for the *i*-th patient during period *k* is defined as follows:

6 $$\begin{aligned} MOTox_{ijk} = \frac{1}{|\mathcal {M}_j|} \sum _{m=1}^{|\mathcal {M}_j|} tox_{ijk}^{m} + \max _{m=1,...,|\mathcal {M}_j|}{\left( tox_{ijk}^{m}\right) }. \end{aligned}$$

Specifically, four different MOTox scores can be computed for each subject.

By adopting this approach rather than relying on individual CTCAE grades for diverse toxicities, problems associated with dealing with a vast number of potential confounder combinations are mitigated. This ensures increased feasibility in the analysis. When considering individual grades for each toxicity, the number of possible confounders combinations would be too high leading to a violation of positivity. Additionally, this approach alignes with clinical practices, where treatment adaptation occurs based on the patient’s overall toxic burden due to the presence of multiple toxicities.

#### Directed acyclic graph (DAG)

**Fig. 2:**
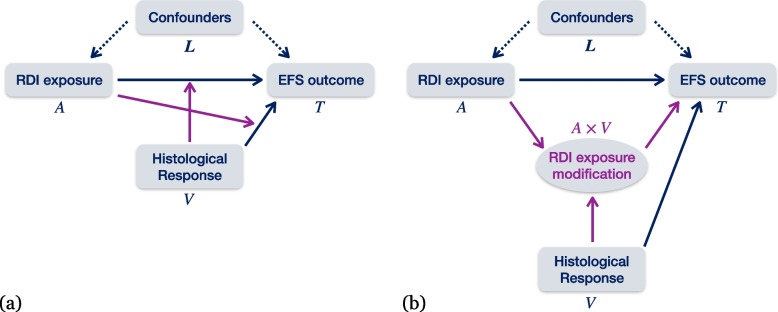
Directed Acyclic Graph (DAG) that represents the causal relationships between EFS outcome (*T*), RDI-exposure (*A*), pre-/post-assignment confounders ($\varvec{L}$), and HRe as effect modifier (*V*). The exposure-effect modification pathway $A\times V \rightarrow T$ (in purple) is depicted in DAG (**a**) using the “arrow-on-arrow" representation proposed by [34], whereas in DAG (**b**) including the additional node with both *A* and *V* as parents, as suggested by [35]

Figure 2 presents two alternative visualizations of the causal structure involving RDI-exposure (*A*), EFS outcome (*T*), pre- and post-assignment confounders ($\varvec{L}$), and HRe as a modifying variable (*V*). In both cases, blue solid arrows indicate that both exposure *A* and the effect modifier *V* directly influence the outcome *T*, while dashed blue arrows represent the confounding relationship between *A* and *T*. The purple arrows represent the influence of exposure-effect modification $A\times V$ on *T*, but there is no unanimous consensus on how to graphically represent $A\times V \rightarrow T$. In this regard, DAG (a) utilizes the crossing “arrow-on-arrow" representation provided by [34], while DAG (b) includes an additional node with both *A* and *V* as parents, as proposed in [35].

The causal structure relies upon the hypothesis that there is no path between HRe and RDI-exposure, i.e., $A {\nleftrightarrow } V$. This assumption is motivated by the following reasons.

**Fig. 3:**
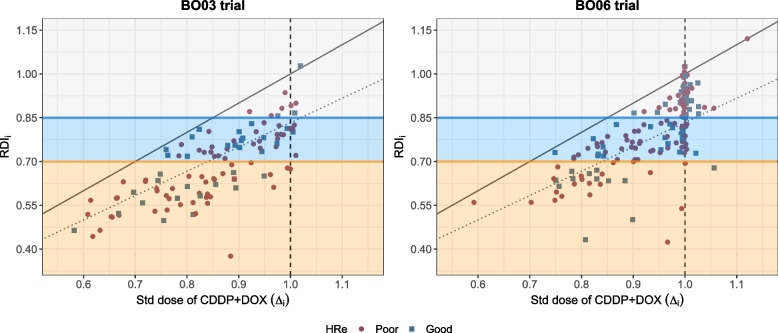
Scatter plots of $RDI_i$ against the standardized dose $\Delta _i$ of CDDP+DOX conditional on trial (left panel: *BO03*; right panel: *BO06*) and HRe (purple points: *poor*; blue squares: *good*)

1. HRe is the result of the histopathological examination after pre-operative chemotherapy. This means that RDI computed as in Eq. (1) at the end of treatment (i.e., after both the pre- and the post-operative periods) could not affect HRe. This was confirmed by the absence of evidence indicating an association between the final RDI and HRe (see Fig. 3), as reported in [18] as well. Therefore $A \nleftrightarrow V$.
2. In the original BO03/BO06 RCTs, HRe was not known until several weeks since chemotherapy is resumed after surgery. This means that HRe result could have influenced the decision to modify therapy only in the last cycles. However, the original RCT protocols did not provide for treatment interventions based on HRe. As clinicians are generally committed to adhering to the planned treatment without being influenced by factors not foreseen in the protocol, very few protocol violations are expected in a RCT. Therefore, $V {\nleftrightarrow } A$.

### Statistical analysis

Once the causal inference framework has been defined, statistical analysis can be performed. This requires careful consideration about the identifiability assumptions [19, 31] related to *positivity* and *absence of model misspecification* (see Supplementary Material B). Building upon the ITT landmark Cox model examined in [6], the idea is to assess subgroup-specific causal effects of different RDI-exposure stategies on EFS-time using a Cox MSM with effect modification. In the ITT model from [6], the analysis incorporated the intended treatment, HRe, and their interaction to investigate the effect of assigned regimens stratified by HRe. In the Cox MSM proposed here, the binary variable representing intended treatment is replaced by two dummy variable representing *reduced* and *highly-reduced* RDI strategies, and their effects are assumed to vary based on the effect modifier (HRe).

#### Marginal structural Cox model with effect modification

Cox MSMs are a class of causal models that focus on *counterfactual* time-to-event variables [19, 36, 37]. These variables represent the time at which an event would have been observed had a patient been administered a specific exposure level *a*, which might differ from the actual treatment received. In our context, the *counterfactual EFS time* that would be observed in a subject under exposure $a \in \{0,1,2\}$ is denoted by $T^{a}$.

The Cox-type marginal structural hazard function for counterfactual EFS time under RDI-exposure $a\in \{0:\textit{standard}; \, 1:\textit{reduced};\, 2:\textit{highly-reduced}\}$ with effect modification given by HRe variable $V \in \{0:\textit{poor};\, 1:\textit{good}\}$ is defined as follows:

7 $$\begin{aligned} h_{T^{a}}(t|V) = h_0(t) \exp \left\{ \beta _1 \mathbbm {1}_{(a=1)} + \beta _2 \mathbbm {1}_{(a=2)} + \beta _3 \mathbbm {1}_{(a=1)}V + \beta _4 \mathbbm {1}_{(a=2)} V + \beta _5 V \right\} . \end{aligned}$$

Additive effect modification is present for a *reduced* RDI if $\beta _3\ne 0$ or for a *highly-reduced* RDI if $\beta _4\ne 0$.

Evidence for effect modification aids in identifying groups of individuals with specific inherent characteristics which make them better responsive to treatment, while in others, treatment may be less effective, ineffective, or even harmful [32].

#### Inverse probability of treatment weighting (IPTW)

To estimate the causal parameters $\varvec{\beta }$ of the Cox MSM defined in (7), a weighted Cox model [38, 39] can be fitted to the pseudo-population obtained through IPTW, as follows:

8 $$\begin{aligned} h^{SW_i }_{T_i}\left( t | A_i, V_i\right) = h_0(t) \exp \left\{ \theta _1 \mathbbm {1}_{(A_i=1)} + \theta _2 \mathbbm {1}_{( A_i=2)} + \theta _3 \mathbbm {1}_{(A_i=1)}V_i + \theta _4 \mathbbm {1}_{(A_i=2)} V_i + \theta _5 V_i \right\} \end{aligned}$$

with subject-specific stabilized weights given by

9 $$\begin{aligned} SW_i= \frac{ P\left( A_i | V_i \right) }{ P\left( A_i \big | \varvec{L}_i, V_i\right) }. \end{aligned}$$

The numerator in (9) represents the probability that a subject *i* received exposure $A_i$ given their HRe $V_i$. Including the effect modifier in the numerator generally results in narrower confidence intervals around the effect estimates [19]. The denominator is the probability that the subject received exposure $A_i$ given HRe and confounders. In this case, the effect modifier is included to enhance the efficiency of the MSM parameter estimation process, as recommended in [19]. Both numerator and denominator are modelled by employing multinomial logistic regression models.

Under causal inference assumptions, association is causation in the pseudo-population and the estimates of the associational parameters $\varvec{\theta }$ are consistent for the causal parameters $\varvec{\beta }$. Nonetheless, a note of caution is required in applying this methodology to the chemotherapy data. Different model specifications in terms of confounding covariate features must be compared to satisfy the final assumptions of *positivity* and *no misspecification of the weight-generating models* (see Supplementary Material B) and guarantee an unbiased estimation of the results. Specifically, a mean weight value that significantly deviates from one or the presence of extreme values in the distribution of the stabilized weights can signal potential issues related to positivity violation or model misspecification [40]. In addition, graphical methods can be employed to check covariate balance between the exposure groups in the weighted samples [41].

#### Conditional average treatment effects (CATEs)

The Restricted Mean Survival Time (RMST) [42, 43] is employed as measure of treatment effect. More precisely, the RMST at time *t* under strategy $a\in \{0,1,2\}$ for individuals in sub-group $v\in \{0,1\}$ is the expected conditional time-to-event defined as follows:

10 $$\begin{aligned} \mu _a(t;v) = \mathbbm {E}[\text {min}\{T^a,t\} | V=v] = \int _0^{t} S^a(s| V=v)ds. \end{aligned}$$

This corresponds to the area under the counterfactual survival curve given the effect modifier *V* truncated at time *t*.

The Conditional Average Treatment Effect (CATE) at time *t*, or the “benefit" in each HRe sub-group, is measured as the contrast between the RMSTs of an RDI-reduction intervention ($a={1,2}$) and the *standard* strategy, as follows:

11 $$\begin{aligned} \tau _{a}(t;v) = \mu _{a}(t;v) - \mu _{0}(t;v) \qquad a\in \{1,2\}, v\in \{0,1\}. \end{aligned}$$

CATE is hence an estimate of the average months gained (if $>0$) or lost (if $<0$) at time *t* by employing RDI-reduction strategy $a\in \{1,2\}$ in sub-group $V=v$.

#### IPTW-based bootstrap procedure for estimating confidence intervals for CATEs

To construct 95% point-wise Confidence Intervals (CIs) for each CATE, a generalized bootstrap procedure is proposed. This novel sampling procedure differs from typical random sampling by (i) separately considering the sub-cohorts defined by different combinations of strategies and effect modifier levels, (ii) utilizing unequal probability sampling [22, 23] based on estimated IPTW stabilized weights, and (iii) sampling (with repetitions) from each sub-cohort while maintaining sub-sample sizes. At each iteration the generalized bootstrap sample is generated as the union of the various bootstrap sub-samples. The steps are detailed as follows.

1. Determine the set of possible sub-cohorts:
  $$\begin{aligned} \mathcal {G} = \left\{ (a,v):\, a=0,1,2;\, v=0,1\right\} \end{aligned}$$
2. Assign the subjects to the sub-cohorts $g\in \mathcal {G}$:
  $$\begin{aligned} \mathcal {D}_g = \left\{ i \in \{1,\dots ,N\}: (A_i, V_i)=g\right\} \quad \text { with sample size } n_g = |\mathcal {D}_g|. \end{aligned}$$
3. For each sub-cohort $g\in \mathcal {G}$, compute the sampling probability of each subject $j\in \mathcal {D}_{g}$ as a transformation of their stabilized weight from IPTW-Eq. (9) as follows:
  $$\begin{aligned} p_{gj} = \frac{sw_j}{\sum\nolimits_{k=1}^{n_{g}} sw_k}. \end{aligned}$$
  These unequal sampling probabilities represent the normalized IPTW stabilized weights within the sub-cohort *g* in such a way that $\sum _{j\in \mathcal {D}_{g}} p_{gj} = 1$.
4. At each bootstrap iteration $b=1,\dots , B$ (with $B=1000$):
  - obtain the sub-samples $\mathcal {D}^b_{g}$ with $n_g$ subjects sampled with repetitions from $\mathcal {D}_{g}$, where each subject *j* has probability $p_{gj}$ to be selected;
  - combine the sub-samples $\mathcal {D}_{g}^b$ into the generalized bootstrap sample $\mathcal {D}^b$:
    $$\begin{aligned} \mathcal {D}^b = \bigcup _{g \in \mathcal {G}} \mathcal {D}_g^b \quad \text { where } \quad |\mathcal {D}^b| = \sum _{g \in \mathcal {G}} n_g = N; \end{aligned}$$
  - Estimate the CATEs $\tau _{a}^b(t;v)$ over time *t* in (11) on the generalized bootstrap sample $\mathcal {D}^b$.
5. For each RDI-reduction strategy $a\in \{1,2\}$, effect modifier stratum $v\in \{0,1\}$ and time-point *t*, the estimates $\hat{\tau }_{a}^b(t;v)$ are ordered from smallest to largest. The resulting 2.5th and 97.5th percentiles are selected to define the bounds of the 95% bootstrap CI [20, 21].

## Results

Statistical analyses were performed in the R-software environment [44], in particular using ipw [45] and survival [46] packages. Source code for the current study is available here: https://github.com/mspreafico/TTEcausalRDI.

### Study cohort

In total 444 eligible patients were enrolled in the control arms of BO03 (199) and BO06 (245). In this sample, 106 (23.9%) patients were excluded due to missing HRe. Among the remaining 338 patients, 58 subjects stopped the chemotherapy treatment or did not undergo surgery, while 4 completed the treatment but experienced an event during its administration. The final cohort of 276 patients who successfully completed the standard EOI treatment (114 from BO03 and 162 from BO06, respectively) included in the per-protocol analyses (62.2% of the initial sample) is shown in the consort diagram in Supplementary Material C.

### Descriptives

Patient characteristics over the entire cohort and by trial are shown in Table 2. Overall, the median RDI value was 0.759 (IQR=[0.649; 0.857]), with minimum and maximum values of 0.376 and 1.121. This corresponded to a total of 75 patients (27.2%) with *standard* RDI, 111 (40.2%) with *reduced* RDI, and 90 (32.6%) with *highly-reduced* RDI. Median EFS time computed using the reverse Kaplan-Meier method [47] was 89.59 months (IQR = [50.33; 146.30]) and 152 patients (55.1%) experienced an event after the end of the therapy. *Generic* MOTox scores were high: pre/post-operative median MOTox values were equal to 4.5; this means that in median patients experienced at least one generic side effect of CTCAE-grade 3 (i.e., severe or medically significant). This is not surprising because nausea is the most common chemotherapy-induced adverse event. *Rule-specific* MOTox resulted higher in the post-operative period than in the pre-surgery one. This indicated that toxicity levels accumulate over time resulting in a more severe overall toxic burden in the second phase of treatment. A total of 94 patients (34.1%) experienced *good* HRe after surgical resection.

Figure 3 shows a scatter plot of $RDI_i$ against the standardized dose $\Delta _i$ of CDDP+DOX for each trial (left panel: *BO03*; right panel: *BO06*) and HRe (circles: *poor*; squares: *good*). Points to the left of the black dashed vertical line, where $\Delta _i<1$, represent patients who received dose-reduced therapies. The black diagonal solid line satisfies equation $RDI_i = \Delta _i$, dividing the group of patients with standardized time $\Gamma _i>1$ (delayed therapy, below the line) from the group of patients with $\Gamma _i<1$ (anticipated therapy, above the line). The black diagonal dotted line satisfies equation $RDI_i = \Delta _i/1.2$, dividing the group of patients with therapy delayed by more than 20% of anticipated time (below the dotted line) from the group of patients with therapy delayed by less than 20% of anticipated time (between solid and dotted black lines). Solid horizontal lines vertically divide patients with a *standard* RDI-exposure (gray area above the blue line) from those with *reduced* (blue area between the blue and orange lines) and *highly-reduced* exposure (orange area below the orange line). This figure shows lack of a clear association between HRe and RDI-exposure, as confirmed by the chi-squared test (*p*-value = 0.614).

### IPTW diagnostics

Multinomial logistic regressions were used to model both numerators and denominators of stabilized weights $SW_i$ in (9). Five different IPTW specifications in terms of the confounding covariates included in the denominators (Table 3) were investigated to determine whether and which models best satisfied *positivity* and *no misspecification*. See Supplementary Material D for further details.

**Table 3:** Inverse Probability of Treatment Weighting (IPTW) diagnostics based on summaries of stabilized weights $SW_i$ by different specifications of multinomial logistic regressions for the denominator $\Pr \left( A_i \big | \varvec{L}_i\right)$

IPTW Specification		Stabilized weights: $\varvec{SW}_{\varvec{i}}$	
*Method*	*Description*^a^	*Mean (s.d.)*	*Min/Max*
IPTW 1	Categorical/binary confounders and binary effect modifier as main effect only; each continuous MOTox score linearly related to the log-odds.	0.988 (0.663)	0.311/5.154
IPTW 2	Same as in IPTW 1 + interaction terms for toxicity confounders linearly related to the log-odds.	0.989 (0.691)	0.334/5.102
IPTW 3	Same as in IPTW 1 + interaction terms between toxicities and trial linearly related to the log-odds.	0.977 (0.685)	0.305/5.245
IPTW 4	Categorical confounders and effect modifier as main effect only; B-spline basis matrix for cubic polynomial splines with three internal knots were used to model the relationship between each continuous MOTox score and the log-odds.	0.969 (0.813)	0.306/7.027
IPTW 5	Same as in IPTW 1 + interaction terms between toxicities and histological response linearly related to the log-odds	0.963 (0.694)	0.279/8.386

^a^See Supplementary Material D for further details

**Fig. 4:**
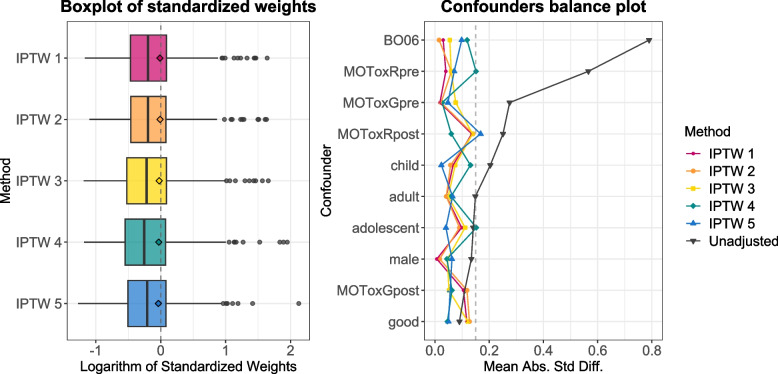
Diagnostic plots for Inverse Probability of Treatment Weighting (IPTW) performed by using the five different specification methods in Table 3 (purple: *IPTW 1*; orange: *IPTW 2*; yellow: *IPTW 3*; green: *IPTW 4*; blue: *IPTW 5*). Left panel: Boxplots of subject-specific stabilized weights $SW_i$ computed via Eq. (9) in logarithmic-scale. Diamonds represent the mean values. Right panel: Confounder balance plot. Lines represent the mean absolute standardized differences for each exposure-related confounder according to the four different specification methods (colored lines) and their unadjusted version (black line)

The distributions of the stabilized weights (Table 3; left-panel of Fig. 4) suggest that there was no evidence of violation of the positivity or misspecification assumptions for IPTW methods 1 and 2 (mean values of about 0.99 without extreme values), whereas methods 3 to 5 presented lower mean values and higher standard deviations. The same was confirmed by the diagnostics balance plot in the right panel of Fig. 4. The mean absolute standardized differences for confounders in the unweighted sample (black points) always exceeded those in the weighted samples, and the lowest values were observed for IPTW 1 and 2. IPTW model 1 was finally selected due to the lower number of parameters.

### Estimated causal effects

The causal parameters $\varvec{\beta }$ in Cox MSM (7) were estimated through their consistent parameters $\varvec{\theta }$ in weighted Cox model (8) fitted on the pseudo-population obtained with IPTW 1. Robust standard errors for computing the confidence interval of each coefficient were obtained via the option robust=TRUE in R function coxph [46]. Estimates were finally compared to the results obtained by fitting a traditional Cox model [14] on the original unweighted population.

Table 4 shows the results in the pseudo-population (parameters $\hat{\varvec{\beta }}$ to the left) and the original population (parameters $\varvec{\hat{\beta }}_{\text {unw}}$ to the right). The difference clearly demonstrates how the latter was affected by the toxicity-treatment-adjustment bias. RDI exposure in the original population was not randomized: the final RDI represented the realization of the treatment trajectory influenced by both the severity of overall toxicity experienced by each patient and physicians’ interventions. In the pseudo-population mimicking the TT, randomization was emulated by adjusting for confounding. Therefore, the results can be interpreted in a causal setting.

**Table 4:** Estimated parameters $\hat{\varvec{\beta }}$ along with their 95% Confidence Intervals (CIs) for the Cox MSM in Eq. (7) and for the corresponding unweighted traditional Cox model

	Cox MSM		Unweighted Cox model	
	*Pseudo-population*		*Original population*	
Covariate	$\hat{\varvec{\beta }}$	*95% CIs*	$\hat{\varvec{\beta }}_{\textbf{unw}}$	*95% CIs*
$a=1$	$-0.536$	$[-1.031; -0.041]$	$-0.116$	$[-0.568; 0.335]$
$a=2$	$-0.808$	$[-1.384; -0.231]$	$-0.359$	$[-0.844; 0.127]$
$a=1 \times V=1$	0.747	$[-0.379; 1.873]$	$-0.006$	$[-0.997; 0.984]$
$a=2 \times V=1$	1.747	[0.554; 2.939]	0.979	[0.035; 1.923]
$V=1$	$-1.909$	$[-2.835; -0.981]$	$-1.175$	$[-1.921; -0.429]$

**Fig. 5:**
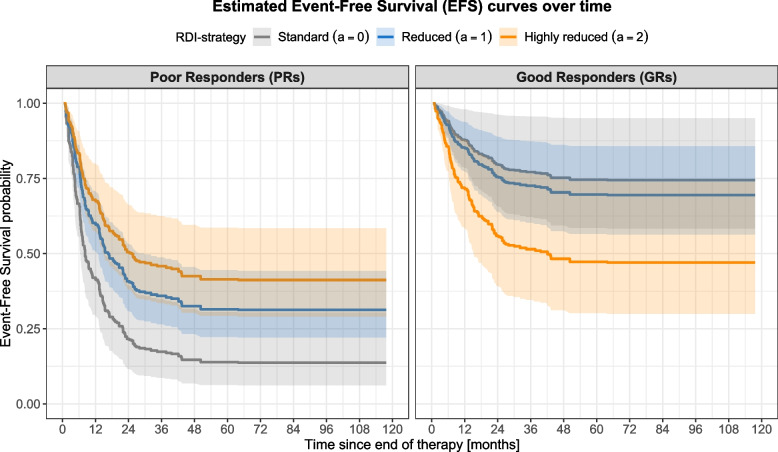
Estimated Event-Free Survival (EFS) over time $\hat{S}^{a}(t|V=v)$ over time (up to 10 years since end of therapy) for *standard* (gray: $a=0$), *reduced* (blue: $a=1$), and *highly-reduced* RDI (orange: $a=2$) strategies in subgroups of Poor Responders (PRs) (left panel: $v=0$) and Good Responders (GRs) (right panel: $v=1$)

Figure 5 displays the estimated EFS curves $\hat{S}^{a}(t|V=v)$ over time (up to 10 years since end of therapy) for *standard* (gray), *reduced* (blue), and *highly-reduced* (orange) RDI-strategy across *poor* (left panel) or *good* (right panel) responders. Results indicate evidence of effect modification: exposures characterized by lower RDI resulted in better EFS in PRs, while, conversely, lower RDI-exposure led to poorer EFS in GRs.

**Fig. 6:**
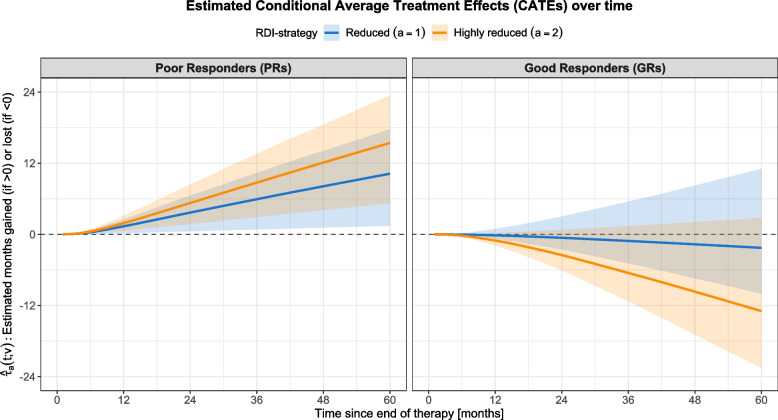
Estimated Conditional Average Treatment Effects (CATEs) $\hat{\tau }_{a}(t;v)$ over time (up to 5 years since end of therapy) along with 95% bootstrap-percentiles CIs for *reduced* (blue: $a=1$) and *highly-reduced* RDI (orange: $a=2$) strategies compared to *standard* in subgroups of Poor Responders (PRs) (left panel: $v=0$) and Good Responders (GRs) (right panel: $v=1$)

Figure 6 shows the estimated CATEs over time (up to 5 years since end of therapy) for *reduced* (blue) and *highly-reduced* (orange) RDI-strategy (compared to *standard*) across patients with *poor* or *good* HRe, along with the estimated 95% bootstrap CIs. The CATE trends differ between PR and GR subgroups due to the heterogeneous effect of RDI reductions. GRs (right panel) exhibited a trend towards a clinical disadvantage resulting from reduced RDI, especially for *high-reduced* strategy. Conversely, PRs (left panel) showed a clinically relevant benefit from reducing RDI, meaning that the intrinsic nature of PRs induced resistance to chemotherapy. In particular, 5-year estimated CATEs ($t=60$ months) were $\hat{\tau }_{1}(60;0)=10.2$ (95% $CI = [1.5,17.7]$) and $\hat{\tau }_{2}(60;0)=15.4$ (95% $CI = [5.2,23.5]$), indicating an average gain of 10.2 and 15.4 months for *reduced* and *highly-reduced* exposure, respectively.

### Clinical interpretation

A clinical explanation for these findings may lie in the effect of chemotherapy on non-cancerous cells. Chemotherapy targets a broad spectrum of rapidly dividing cells, including immune cells, which are essential for detecting and destroying cancerous cells and protecting the body from infections. Damage to these immune cells impairs the immune system’s processes and ability to fight cancer, leading to a weakened response and compromising the body’s natural defense against any remaining cancerous cells.

In patients who responded well to treatment, chemotherapy’s effectiveness in reducing tumor burden outweighed its negative effects, leaving fewer cancer cells for the immune system to handle, partially offsetting immune suppression. In contrast, in PRs, chemotherapy was less effective in directly targeting the tumor, and its damaging impact on the immune system was more pronounced. In such cases, an increased RDI may have led to a weakened and further suppressed immune response, amplifying the imbalance between the positive and negative effects of chemotherapy, and resulting in poorer EFS outcomes.

## Discussion

Motivated by a sharp yet delicate clinical question, this paper introduces a novel approach to mimic a hypothetical target trial using RCT data with interventions. The final aim was to investigate the effect of reductions in RDI on EFS in patients with osteosarcoma, with a focus on subgroups of poor and good responders. Chemotherapy administration data in osteosarcoma from BO03 and BO06 RCTs were analysed. IPTW was first used to transform the original selected population into a pseudo-population emulating the randomized cohort of the TT. Then, Cox MSM with effect modification was employed to compared the effects of RDI reductions ranging from 15% to 30% (*reduced* exposure) or above 30% (*highly-reduced* exposure) to the *standard* RDI of EOI tratment (structured in 6 cycles of 3-weekly CDDP+DOX) in both PRs and GRs. CATEs were finally measured as the contrast between the RMST of *reduced/highly-reduced* RDI-strategy and that of the *standard* one. The 95% CIs for CATEs were obtained using a novel IPTW-based bootstrap procedure while preserving the sizes of sub-cohorts.

### Complexity of chemotherapy data and causal assumptions

Considering the data complexity and the underlying causal assumptions, a note of caution is required, as it needs to encompass all aspects of the chemotherapy process. First, exposure and outcome must be properly defined to guarantee the consistency assumption. Then, pre- and post-assigned confounders must be carefully identified to satisfy the assumption of no unmeasured confounding. During this process, it is imperative to notice that assignment of dose reductions or delays in chemotherapy administration was determined not by individual toxicities but by the overall toxic burden for each patient. Therefore, pre- and post-operative side-effects data were summarized using the new Multiple Overall Toxicity (MOTox) approach [25] for both *rule-specific* and *generic* toxicity. This novel analytical strategy allowed (i) to reduce the number of possible confounders combinations dealing with non-positivity and highly-correlated data, and (ii) to meet the clinical rationale of tailoring treatment according to the patient’s overall toxic burden in the presence of multiple toxic side effects. Third, different weighting models have to be compared in order to preserve positivity and guarantee a correct IPTW specification. Finally, an outcome model to address the research question at hand must be correctly specified. This led to the definition of a Cox MSM with effect modification given by HRe, which represents the causal RDI analogue of the ITT landmark Cox model presented in [6]. These steps required careful consideration and were crucial for ensuring unbiased results of the per-protocol analysis.

### Study contributions

The first significant contribution of this study is its innovative use of TT emulation to address the research question. The results revealed evidence for effect modifications by HRe, as increasing RDI-reductions caused two opposite trends for PRs and GRs. Specifically, higher RDI reductions led to improved EFS in PRs but worsened EFS in GRs. Estimated CATEs highlighted that PRs can significantly benefit from reduced RDI, due to their intrinsic resistance to chemotherapy.

Furthermore, the proposed TT emulation approach enabled the identification of potential pitfalls in a naive RDI-based analysis of chemotherapy data. When the ITT model from [6] was adapted into the traditional Cox models fitted on the unweighted original population by neglecting the influence of toxicities or other confounding factors, the results were influenced by the presence of the *toxicity-treatment-adjustment* bias. By employing an IPTW-based Cox MSM, this study eliminated the feedback loop between side effects and treatment adjustments. This resulted in unbiased estimates of the impact of RDI reductions on EFS within the two subgroups and provided a more accurate depiction of the effects of low-intensity regimens.

The third significant contribution of our study is to have demonstrated how existing RCT data can be effectively repurposed for additional retrospective analyses, extending beyond the intended scope of the original studies. Our proposed analytical approach possesses the versatility to be adapted and applied to various cancer-related investigations. These investigations might encompass diverse types of treatments (e.g., immunotherapies or molecularly targeted agents) with its unique set of side effects to study how reductions in treatment intensity influence the outcome of interest, possibly within specific subgroups of interest. This would require a detailed protocol and close collaboration with medical staff to identify patient’s clinical history, relevant side effects, and treatment-related factors.

Finally, a novel generalized bootstrap procedure was introduced to compute confidence intervals for CATEs. This approach diverged from typical random sampling in two key ways: (i) it sampled from each sub-cohort characterized by various combinations of strategies and effect modifier levels while maintaining the sub-sample sizes, and (ii) it employed unequal IPTW-based probability sampling [22, 23]. By employing this procedure, all sub-cohorts were adequately represented in the generalized bootstrap samples, avoiding estimation issues due to missing observations. Moreover, subjects with oversized weights were sampled more frequently, allowing for a more thorough exploration of the uncertainty associated with them [23]. This approach heavily relies on the estimated stabilized weights. Therefore, it is crucial that the theoretical assumption of no unmeasured confounding holds, and the IPTW model must be correctly specified.

### Limitations

As the first study to use TT emulation to assess whether reduced RDI led to improved EFS, this analysis has certain limitations. Due to the nature of the toxicity data in the BO03 trial, only the highest CTCAE grades for each toxicity during pre- and post-operative cycles were measured. Consequently, the MOTox scores in (6) were based on pre- and post-operative periods rather than being computed per cycle, thus flattening the toxicity history. This data-forced approach is debatable, as multiple severe toxicities might occur simultaneously, potentially leading to significant interactions, and longer-lasting lower-grade toxicities may also impact patient outcomes. However, there is no certainty that the measured highest CTCAE grades happened simultaneously, and longer-lasting lower-grade events may have been obscured by a single severe episode. These aspects could not be adequately addressed in our context, and the approach adopted was the best possible option given the available data.

Upon clinical request, the study investigated patients who had successfully completed standard EOI treatment. Consequently, the analysis was limited to cohort members who underwent surgery and all six cycles of chemotherapy. This corresponded to performing a per-protocol analysis analogous to TT on the RCT data (see Table 1), where EFS was measured from the end of therapy. The actual time between study enrollment and the end of therapy was included in the computation of the RDI level. Extending the proposed TT emulation approach to the entire cohort would require adapting the methodology and the causal estimands to address: (i) time-varying exposure strategies (as RDI level may vary over cycles) and confounders; (ii) intermittent missing exposures (e.g., dosages) or characteristics (e.g., toxicity, effect modifiers) between cycles; (iii) informative dropouts (e.g., treatment discontinuation due to excessive toxicity); (iv) censoring or death before the end of therapy. These extensions require the development of complex methodologies that integrate TT emulation into a longitudinal causal framework for chemotherapy data, while also addressing missing values and within-treatment censoring. Fulfilling the causal assumptions and estimating causal models may be even more demanding in a longitudinal context, representing an interesting and valuable methodological challenge for future research.

### Clinical impact and future research directions

This study revealed that exposures characterized by higher RDI resulted in poorer EFS outcomes in PRs, probably due to a weakened immune response due to chemotherapy effects on non-cancerous cells, combined with insufficient tumor reduction. In contrast, in GRs, the efficacy of chemotherapy in reducing tumour burden outweighed its negative effects on immune suppression. This evidence for effect modification can be exploited for establishing new treatment guidelines tailored to specific patient subgroups that could benefit from modified treatment strategies. While treatment decisions for GRs could be made on a case-by-case basis, as their situation is less severe, guidelines for PRs should recommend treatment strategies that preserve immune function. This could be done by reducing the RDI or combining chemotherapy with immune-supportive therapies. Future clinical studies should further investigate this phenomenon and its translation into clinical practice.

This work also emphasizes the importance of meticulous attention to the collection of chemotherapy data. Data from existing RCT can be used for further retrospective analyses beyond their original purpose only if they are of high quality. High-quality data are indeed essential for developing causal models that accurately account for the complex interactions between chemotherapy and toxicity, thereby minimizing the risk of biased results. This aspect must be carefully considered when designing case report forms for data collection in future trials.

Overall, this study provides a solid foundation for future research toward a cycle-by-cycle longitudinal perspective. Although this methodological extension would require developing complex methods to fully capture the peculiarities of cancer dynamics, it holds significant potential for shedding light on complex aspects of toxicity-treatment interactions. By advancing methods in this research field and collaborating with medical personnel, the results could generate new knowledge in cancer treatment. For example, a novel longitudinal methods to investigate whether treatment delays and dose reductions recommended by guidelines can be accepted without compromising outcomes could be valuable for guiding treatment decisions and optimizing patient care. This approach may also significantly impact the formulation of new guidelines and their implementation in practice.

## Conclusions

This work has introduced an innovative and comprehensive analysis of chemotherapy administration RCT data, aimed at addressing specific clinical questions related to the reduction of RDI within subgroups of patients with osteosarcoma. The study is complemented by tutorial-like explanations which provide insights into the inherent challenges in this scenario and the novel problem-solving strategies proposed. Furthermore, this study has emphasized the critical role of toxicities in this context and illustrated the detrimental consequences of neglecting them in the analyses.

To the best of our knowledge, no other studies have employed the principle of TT emulation to address this important research question in the oncological field. The developed approach offers several advantages, including (i) accounting for all the unique aspects of chemotherapy, (ii) mitigating the *toxicity-treatment-adjustment* bias, and (iii) effectively repurposing existing RCT data for additional retrospective analyses extending beyond the intended scope of the original trials.

This study provides a solid foundation for future work toward a cycle-by-cycle longitudinal perspective, which could significantly enhance cancer research by capturing the complexities of cancer dynamics and chemotherapy data across treatment cycles.

## Supplementary Information

Supplementary Material 1: Supplementary Material A describes the classification of severity for *rule-specific* and *generic* toxicities according to the Common Terminology Criteria for Adverse Events Version 3 [27]. Supplementary Material B discusses the identifiability assumptions for causal inference through MSMs. Supplementary Material C reports the flowchart of cohort selection. Supplementary Material D describes the various denominator models investigated for IPTW.

## Data Availability

Data are not publicly available due to privacy restrictions. Access to the full datasets can be requested to MRC Clinical Trials Unit at UCL, Institute of Clinical Trials and Methodology, UCL, London.

## Abbreviations

CI: Confidence interval
CDDP: Cisplatin
CTCAE: Common terminology criteria for adverse events
DAG: Directed acyclic graph
DOX: Doxorubicin
EOI: European osteosarcoma intergroup
EFS: Event-free survival
GRs: Good responders
HRe: Histological response
IPTW: Inverse probability of treatment weighting
ITT: Intention-to-treat
MOTox: Multiple overall toxicity
(Cox) MSM: Marginal structural (Cox) model
PRs: Poor responders
RCT: Randomized clinical trial
RDI: Received dose intensity
TT: Target trial
