# Supplementary material for: Causal effect of chemotherapy received dose intensity on survival outcome: a retrospective study in osteosarcoma
Source: BMC Med Res Methodol. 2024 Dec 3;24:296. doi: 10.1186/s12874-024-02416-x (PMC11613923; doi:10.1186/s12874-024-02416-x)
Supplement: Supplementary file 1 — Supplementary Material 1: Supplementary Material A describes the classification of severity for rule-specific and generic toxicities according to the Common Terminology Criteria for Adverse Events Version 3 [27]. Supplementary Material B discusses the identifiability assumptions for causal inference through MSMs. Supplementary Material C reports the flowchart of cohort selection. Supplementary Material D describes the various denominator models investigated for IPTW. [file 12874_2024_2416_MOESM1_ESM.pdf]

Causal effect of chemotherapy received dose intensity on survival outcome: a retrospective study in osteosarcoma

M. Spreafico*, F. Ieva, M. Fiocco

*Corresponding author: m.spreafico@math.leidenuniv.nl

Supplementary Material

A CTCAE grades specification for ***rule-specific*** and ***generic*** toxicities

In both BO03 and BO06 studies, toxic side effects were recorded using the Common Terminology Criteria for Adverse Events Version 3 (CTCAE v3.0) [1], with grades ranging from 0 (none) to 4 (life-threatening). Table S1 reports the CTCAE-grades for *rule-specific* toxicities (i.e., *leucopenia*, *thrombocytopenia*, *oral mucositis*, *ototoxicity*, *cardiotoxicity* and *neurotoxicity*) and *generic* ones (i.e., *nausea/vomiting* and *infections*).

B Assumptions for causal inference through marginal structural models

The four main assumptions for causal inference with Marginal Structural Models (MSMs) through Inverse Probability of Treatment Weighting (IPTW) are here discussed.

Exchangeability (or conditional exchangeability) implies the well-known assumption of *no unmeasured confounding* [2]. It states that exposure allocation is independent of the potential outcomes conditional on confounders [2], that is:

$$T^a \perp\!\!\!\perp A|\boldsymbol{L}.$$

Table S1 Toxicity coding based on Common Terminology Criteria for Adverse Events (CTCAE) v3.0 by [1] for *rule-specific* (i.e., leucopenia, thrombocytopenia, oral mucositis, ototoxicity, cardiotoxicity and neurotoxicity) and *generic* (i.e., nausea/vomiting and infections) toxicities.

**Toxicity**	**Grade 0**	**Grade 1**	**Grade 2**	**Grade 3**	**Grade 4**
Rule-specific					
Leucopenia	$\geq 4.0 \times 10^9/L$	$[3.0 - 4.0) \times 10^9/L$	$[2.0-3.0) \times 10^9/L$	$[1.0 - 2.0) \times 10^9/L$	$< 1.0 \times 10^9/L$
Thrombocytonpenia	$\geq 100 \times 10^9/L$	$[75 - 100) \times 10^9/L$	$[50 - 75) \times 10^9/L$	$[25 - 50) \times 10^9/L$	$< 25 \times 10^9/L$
Oral Mucositis	No change	Soreness or erythema	Ulcers: can eat solid	Ulcers: liquid diet only	Alimentation not possible
Cardiac toxicity	No change	Sinus tachycardia	Unifocal PVC[1] arrhythmia	Multifocal PVC[1]	Ventricular tachycardia
Ototoxicity	No change	Slight hearing loss	Moderate hearing loss	Major hearing loss	Complete hearing loss
Neurological toxicity	None	Paraesthesia	Severe paraesthesia	Intolerable paraesthesia	Paralysis
Generic					
Nausea/Vomiting	None	Nausea	Transient vomiting	Continuative vomiting	Intractable vomiting
Infection	None	Minor infection	Moderate infection	Major infection	Major infection with hypotension

[1]PVC = Premature Ventricular Contraction

In the absence of randomization, as is the case in observational studies or RCTs with interventions, it is not possible to test for exchangeability. In such situations, expert knowledge becomes essential for identifying an adequate set of joint predictors of exposure and outcome. These predictors should be chosen in a way that, within their respective levels, any associations between exposure and outcome resulting from shared underlying causes are effectively controlled for [2].

Consistency means that the outcome observed for each individual is the counterfactual outcome under the observed treatment history, that is:

$$T^a = T_i \quad \text{for every individual } i \text{ with } A_i = a.$$

This assumption is violated in the presence of misclassification bias [3] and has two requirements [4]:

i. the exposure must be properly defined so that the counterfactual outcomes are well-defined (this implies that a specific exposure may be hypothetically assigned to a subject exposed to a different level);
ii. a link between counterfactuals and observed data is reasonable in the context under study (this means that the equality should be valid for at least some individuals).

Although consistency can not be empirically verified, it is assumed to be plausible in (observational) studies of medical treatments, since it may be possible to change an individual's treatment status [5].

Positivity states that there is a non-zero (i.e., positive) probability of receiving every level of exposure for every combination of covariates that occur among individuals in the population [2]. In the context under analysis, this corresponds to

$$\Pr\left(A_i = a \middle| \boldsymbol{L}_i = \boldsymbol{l}, V_i = v\right) > 0 \quad \forall a, \boldsymbol{l}, v.$$

If this assumption is violated, then the weights in IPTW in Manuscript Equation (9) are undefined leading to biased estimates of the causal effect.

If a subject cannot be exposed to one or more levels of the confounders (e.g., it cannot be treated in the presence of recommendations from guidelines or established contraindications), then positivity is violated due to a *structural* zero probability of receiving the specific exposure. A solution is to restrict the inference to the subset with a positive probability of exposure, whenever possible [5]. Even in the absence of structural zeros, *random* zeros may occur by chance due to small sample sizes or highly stratified data by numerous confounders. The inclusion of weak or highly-stratified confounders can provide a better confounding adjustment but may cause severe non-positivity, increasing the bias and variance of the estimated effect. An indication of non-positivity may be the presence of estimated weights with the mean far from one or very extreme values [5].

No misspecification of both weighting and outcome models means that both the weighting model for IPTW and the structural outcome model, which links

the outcome to the exposure history, must be correctly specified. This assumption has similar roots in all statistical models [3], as model misspecification leads to instability in the Cox MSM estimates [6, 7].

Since the presence of estimated stabilized weights with the mean far from one or with extreme values suggest possible violation of positivity or misspecification of the weight model [5], proper model specifications can be checked by exploring the distribution of weights [2]. In addition, quantitative (e.g., weighted standardized difference to compare means or prevalences) and qualitative graphical methods can be used to assess whether measured covariates are balanced between treatment groups in the weighted sample [8].

C Flowchart BO03/BO06 cohort selection

Figure S1 displays the consort diagram related to the final cohort of 276 patients (114 and 162 from BO03 and BO06, respectively) included in the analyses.

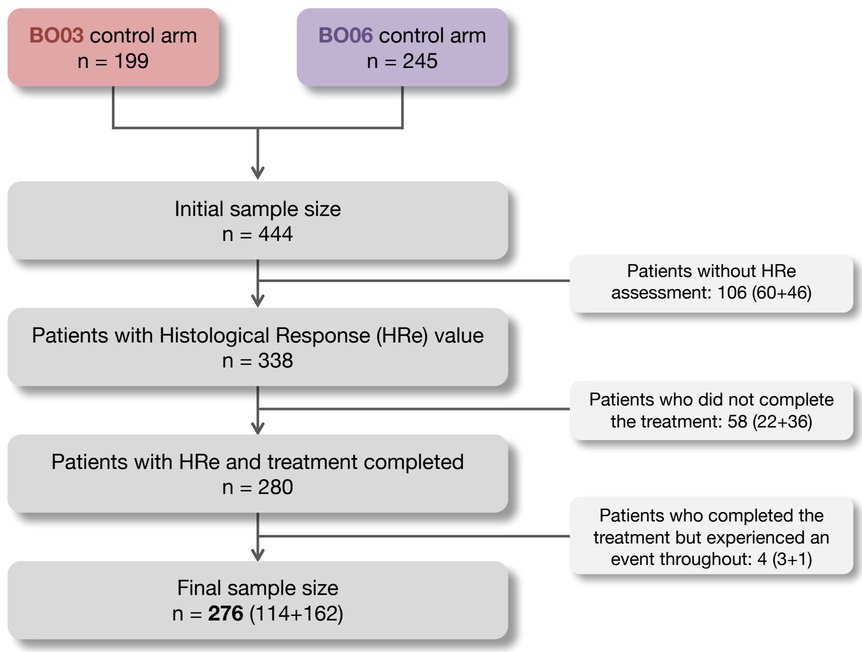

Fig. S1 Flowchart of cohort selection.

D Examining denominator models for IPTW

Different specifications of the subject-specific stabilized weights are investigated in order to check whether and which models best satisfied *positivity* and *no misspecification*. Multinomial logistic regression models are used for both numerators and denominators of SW_i in Manuscript Equation (9).

The numerators $\Pr(A_i|V_i)$, i.e., the probability that a subject i received exposure A_i given his/her histological response V_i, are modelled as:

$$\Pr\left(A_i = a\middle|V_i\right) = \frac{\exp\left(\alpha_{0a} + \alpha_{1a} \cdot \mathtt{GR}_i\right)}{1 + \sum_{j=1}^{2} \exp\left(\alpha_{0j} + \alpha_{1j} \cdot \mathtt{GR}_i\right)} \qquad a = 1, 2;$$
$$\Pr\left(A_i = 0\middle|V_i\right) = \frac{1}{1 + \sum_{j=1}^{2} \exp\left(\alpha_{0j} + \alpha_{1j} \cdot \mathtt{GR}_i\right)},$$

where variable `GR` is the dummy variable for good responders created from categorical variable `HRe` (*poor*; *good*).

The denominator $P\left(A_i\middle|\boldsymbol{L}_i, V_i\right)$ is the probability that the subject received exposure A_i given relative confounders $\boldsymbol{L}_i$ and effect modifier V_i. Five different options are modelled as:

$$\Pr\left(A_i = a\middle|\boldsymbol{L}_i, V_i\right) = \frac{\exp\left(\eta_{ia}\right)}{1 + \sum_{j=1}^{2} \exp\left(\eta_{ij}\right)} \qquad a = 1, 2;$$
$$\Pr\left(A_i = 0\middle|\boldsymbol{L}_i, V_i\right) = \frac{1}{1 + \sum_{j=1}^{2} \exp\left(\eta_{ij}\right)}.$$

by considering five different linear predictors η_{ia} in terms of confounding features.

- **IPTW 1**: categorical/binary confounding covariates and binary effect modifier are included as a main effect only and the MOTox scores are assumed linearly related to the log-odds:

$$\begin{aligned}\eta_{ia} = \gamma_{0a} &+ \gamma_{1a} \cdot \mathtt{BO06}_i + \gamma_{2a} \cdot \mathtt{adolescent}_i + \gamma_{3a} \cdot \mathtt{adult}_i + \gamma_{4a} \cdot \mathtt{male}_i + \\ &+ \gamma_{5a} \cdot \mathtt{MOTox}_{i,gen,pre} + \gamma_{6a} \cdot \mathtt{MOTox}_{i,rule,pre} + \\ &+ \gamma_{7a} \cdot \mathtt{MOTox}_{i,gen,post} + \gamma_{8a} \cdot \mathtt{MOTox}_{i,rule,post} + \gamma_{9a} \cdot \mathtt{GR}_i.\end{aligned}$$

- **IPTW 2**: same as in IPTW 1 + interaction terms for toxicity confounders linearly related to the log-odds:

$$\begin{aligned}\eta_{ia} = \gamma_{0a} &+ \gamma_{1a} \cdot \mathtt{BO06}_i + \gamma_{2a} \cdot \mathtt{adolescent}_i + \gamma_{3a} \cdot \mathtt{adult}_i + \gamma_{4a} \cdot \mathtt{male}_i + \\ &+ \gamma_{5a} \cdot \mathtt{MOTox}_{i,gen,pre} + \gamma_{6a} \cdot \mathtt{MOTox}_{i,rule,pre} + \\ &+ \gamma_{7a} \cdot \mathtt{MOTox}_{i,gen,post} + \gamma_{8a} \cdot \mathtt{MOTox}_{i,rule,post} + \gamma_{9a} \cdot \mathtt{GR}_i + \\ &+ \gamma_{10a} \cdot \mathtt{MOTox}_{i,gen,pre} \cdot \mathtt{MOTox}_{i,rule,pre} + \gamma_{11a} \cdot \mathtt{MOTox}_{i,gen,post} \cdot \mathtt{MOTox}_{i,rule,post}.\end{aligned}$$

- **IPTW 3**: same as in IPTW 1 + interaction terms between toxicities and trial linearly related to the log-odds:

$$\begin{aligned}\eta_{ia} = \gamma_{0a} &+ \gamma_{1a} \cdot \texttt{BO06}_i + \gamma_{2a} \cdot \texttt{adolescent}_i + \gamma_{3a} \cdot \texttt{adult}_i + \gamma_{4a} \cdot \texttt{male}_i + \\ &+ \gamma_{5a} \cdot \texttt{MOTox}_{i,gen,pre} + \gamma_{6a} \cdot \texttt{MOTox}_{i,rule,pre} + \\ &+ \gamma_{7a} \cdot \texttt{MOTox}_{i,gen,post} + \gamma_{8a} \cdot \texttt{MOTox}_{i,rule,post} + \gamma_{9a} \cdot \texttt{GR}_i + \\ &+ \gamma_{10a} \cdot \texttt{trial}_i \cdot \texttt{MOTox}_{i,gen,pre} + \gamma_{11a} \cdot \texttt{trial}_i \cdot \texttt{MOTox}_{i,rule,pre} + \\ &+ \gamma_{12a} \cdot \texttt{trial}_i \cdot \texttt{MOTox}_{i,gen,post} + \gamma_{13a} \cdot \texttt{trial}_i \cdot \texttt{MOTox}_{i,rule,post}.\end{aligned}$$

  This choice is motivated by the statistically different distributions of the MOTox scores in BO03 and BO06 trials (see Manuscript Table 2).

- **IPTW 4**: categorical/binary confounding covariates and binary effect modifier are included as a main effect only; B-spline basis matrix for cubic polynomial splines with three internal knots are used to model the relationship between each continuous MOTox score and the log-odds:

$$\begin{aligned}\eta_{ia} = \gamma_{0a} &+ \gamma_{1a} \cdot \texttt{BO06}_i + \gamma_{2a} \cdot \texttt{adolescent}_i + \gamma_{3a} \cdot \texttt{adult}_i + \gamma_{4a} \cdot \texttt{male}_i + \\ &+ \boldsymbol{\gamma}_{5a}^T \boldsymbol{B}\left(\texttt{MOTox}_{i,gen,pre}\right) + \boldsymbol{\gamma}_{6a}^T \boldsymbol{B}\left(\texttt{MOTox}_{i,rule,pre}\right) + \\ &+ \boldsymbol{\gamma}_{7a}^T \boldsymbol{B}\left(\texttt{MOTox}_{i,gen,post}\right) + \boldsymbol{\gamma}_{8a}^T \boldsymbol{B}\left(\texttt{MOTox}_{i,rule,post}\right) + \gamma_{9a} \cdot \texttt{GR}_i.\end{aligned}$$

- **IPTW 5**: same as in IPTW 1 + interaction terms between toxicities and HRe linearly related to the log-odds:

$$\begin{aligned}\eta_{ia} = \gamma_{0a} &+ \gamma_{1a} \cdot \texttt{BO06}_i + \gamma_{2a} \cdot \texttt{adolescent}_i + \gamma_{3a} \cdot \texttt{adult}_i + \gamma_{4a} \cdot \texttt{male}_i + \\ &+ \gamma_{5a} \cdot \texttt{MOTox}_{i,gen,pre} + \gamma_{6a} \cdot \texttt{MOTox}_{i,rule,pre} + \\ &+ \gamma_{7a} \cdot \texttt{MOTox}_{i,gen,post} + \gamma_{8a} \cdot \texttt{MOTox}_{i,rule,post} + \gamma_{9a} \cdot \texttt{GR}_i + \\ &+ \gamma_{10a} \cdot \texttt{GR}_i \cdot \texttt{MOTox}_{i,gen,pre} + \gamma_{11a} \cdot \texttt{GR}_i \cdot \texttt{MOTox}_{i,rule,pre} + \\ &+ \gamma_{12a} \cdot \texttt{GR}_i \cdot \texttt{MOTox}_{i,gen,post} + \gamma_{13a} \cdot \texttt{GR}_i \cdot \texttt{MOTox}_{i,rule,post}.\end{aligned}$$

  Variable `BO06` is the dummy variable created from categorical variable `trial` (*BO03*; *BO06*). Variables `adolescent` and `adult` are the dummy variables created from categorical variable `age` (*child*; *adolescent*; *adult*). Variable `male` is the dummy variable created from categorical variable `gender` (*female*; *male*).

References

[1] US Department of Health and Human Services. Common Terminology Criteria for Adverse Events v3.0 (CTCAE); 2006. https://www.eortc.be/services/doc/ctc/ctcaev3.pdf.

[2] Cole SR, Hernán MA. Constructing Inverse Probability Weights for Marginal Structural Models. American Journal of Epidemiology. 2008;168(6):656–664. https://doi.org/10.1093/aje/kwn164.

[3] Williamson T, Ravani P. Marginal structural models in clinical research: when and how to use them? Nephrology Dialysis Transplantation. 2017;32(suppl 2):ii84–ii90. https://doi.org/10.1093/ndt/gfw341.

[4] Hernán M, Robins J. Causal Inference: What If. Boca Raton: Chapman & Hall/CRC; 2020.

[5] Cole SR, Frangakis CE. The Consistency Statement in Causal Inference. Epidemiology. 2009;20(3-5). https://doi.org/10.1097/EDE.0b013e31818ef366.

[6] Karim ME, Gustafson P, Petkau J, Yinshan Zhao AS, Kingwell E, Evans C, et al. Marginal Structural Cox Models for Estimating the Association Between β-Interferon Exposure and Disease Progression in a Multiple Sclerosis Cohort. American Journal of Epidemiology. 2014;180(2):160–171. https://doi.org/10.1093/aje/kwu125.

[7] Karim ME, Petkau J, Gustafson P, Tremlett H, Group TBS. On the application of statistical learning approaches to construct inverse probability weights in marginal structural Cox models: Hedging against weight-model misspecification. Communications in Statistics - Simulation and Computation. 2017;46(10):7668–7697. https://doi.org/10.1080/03610918.2016.1248574.

[8] Austin PC, Stuart EA. Moving towards best practice when using inverse probability of treatment weighting (IPTW) using the propensity score to estimate causal treatment effects in observational studies. Statistics in Medicine. 2015;34(28):3661–3679. https://doi.org/10.1002/sim.6607.
